# Mucus Secretion as a Defensive Mechanism in the Freshwater Flatworm *Stenostomum sphagnetorum* Against the Ciliate Predator *Coleps hirtus*

**DOI:** 10.3390/biology14091253

**Published:** 2025-09-12

**Authors:** Gabriele Achille, Santosh Kumar, Daizy Bharti, Graziano Guella, Claudio Ortenzi, Federico Buonanno

**Affiliations:** 1 Laboratory of Protistology and Biology Education, Department of Education, Cultural Heritage, Tourism (ECHT), University of Macerata, 62100 Macerata, Italy; g.achille@unimc.it; 2 Zoological Survey of India, Prani Vigyan Bhawan, M-Block, New Alipore, Kolkata 700 053, India; santoshcbio@gmail.com (S.K.); daizybharti83@gmail.com (D.B.); 3 Bioorganic Chemistry Laboratory, Department of Physics, University of Trento, 38050 Trento, Italy; graziano.guella@unitn.it

**Keywords:** predator–prey interactions, Platyhelminthes, ciliates, rhabdoid glands

## Abstract

This study investigates the defensive mechanisms employed by the freshwater flatworm *Stenostomum sphagnetorum* against the predatory ciliate, *Coleps hirtus*. Focusing on the role of the glandular secretions produced by *S. sphagnetorum*, the research demonstrates that the flatworm secretes mucus that acts as a barrier, disrupting predator attack. In particular, we show that: (1) *S. sphagnetorum* specimens artificially deprived of glandular secretions are significantly more susceptible to predation by *C. hirtus* than untreated specimens; (2) the secretion-deprived organisms consistently exhibit a significantly greater sensitivity to the main toxins employed by *C. hirtus* for predation, relative to untreated counterparts; (3) the characterization of the glandular secretion indicates that the mucus contains both neutral and acidic glycosaminoglycans, along with protein components, suggesting a complex chemical composition that may contribute to its protective function.

## Simple Summary

Predator–prey interactions among microorganisms, including microinvertebrates and protists, exhibit remarkable variability and complexity, often rivaling those observed in vertebrates. Within large Platyhelminthes, many species rely on chemical defenses, frequently involving toxins that deter predators. However, in microturbellarian Platyhelminthes, defensive strategies are less understood. Notably, some species produce a non-toxic mucus secretion believed to function as a defense mechanism, although its mechanism of action remains poorly characterized and nothing is known about its chemical composition. In this study, we investigate the defensive role of the mucus secretion in the freshwater microturbellarian *Stenostomum sphagnetorum* against the predatory ciliate *Coleps hirtus*. Our results confirm that the mucus acts as an effective defensive barrier, physically separating the predator from the prey and neutralizing the predator’s toxic compounds. Preliminary data suggest that the mucus contains a mixture of bioactive substances, which could play synergistic roles in predator avoidance and toxin neutralization.

## 1. Introduction

Catenulid microturbellarians primarily inhabit freshwater ecosystems [1,2] and are known to engage in complex interactions with unicellular eukaryotic organisms both as predators and as prey. One such interaction involves the catenulid *Stenostomum sphagnetorum*, and the ciliated protozoan *Dileptus margaritifer* Dujardin, 1841 [3]. These kinds of interactions highlight the complex ecological relationships between ciliates and small metazoans [4,5,6,7]. Indeed, several ciliates are capable of attacking microturbellarians, as seen with the freshwater prostomatid ciliate *Coleps hirtus* Müller, 1786. *C. hirtus* is a highly versatile predator, feeding on bacteria, algae, flagellates, other ciliates, and animal and plant tissues [8,9]. It uses specialized extrusomes called toxicysts to immobilize and kill its prey by injecting a toxic mixture of bioactive compounds, including 19 saturated, monounsaturated, and polyunsaturated free fatty acids, along with the diterpenoid phytanic acid [9]. This chemical arsenal also enables *C. hirtus* to engage in collective predation, forming large groups to attack bigger organisms or even feed on small vertebrates [10], such as the young larvae of zebrafish (*Danio rerio* Hamilton, 1822) [11] or even its own cells [12].

**Figure 1:**
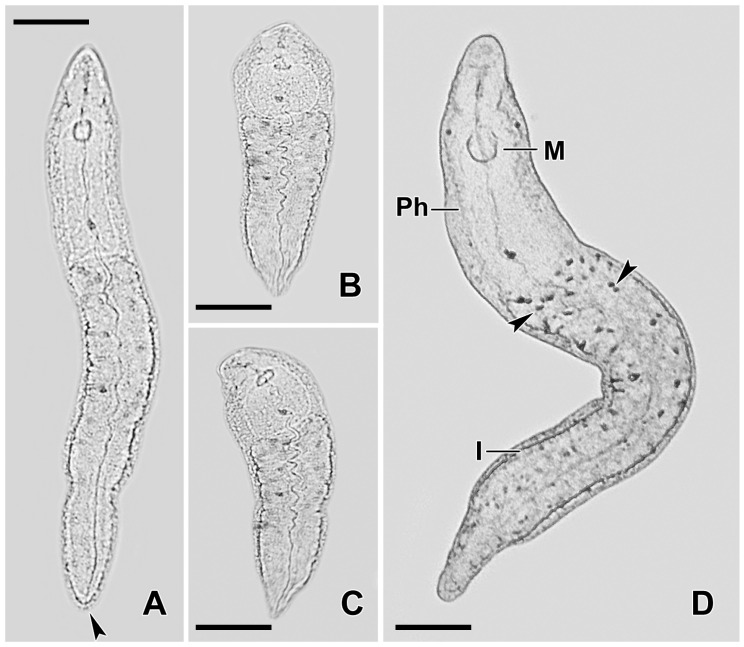
Micrographs of live *Stenostomum sphagnetorum* specimens. (**A**–**C**) Specimens showing different body shapes, with the arrowhead in (**A**) marking the nephridiopore. (**D**) The body shape of a specimen, with arrowheads indicating the excretophores. M, mouth; I, intestine; Ph, pharynx. Scale bars = 100 µm.

In this study, we investigate the predator–prey interaction between *C. hirtus* and the microturbellarian *S. sphagnetorum* (Figure 1), with a particular attention to the defensive strategies employed by the flatworm. It is known that *S. sphagnetorum* can defend itself by secreting a non-toxic mucous material from specialized secretory glands (called rhabdoid glands or false rhabdite glands) [3]. This mucus acts as a physical barrier that can deter the predator, eventually causing it to halt its attack and ingest the mucus. Additionally, the mucus appears to neutralize the toxins released by the predator [3].

Specifically, this study aims to: (1) examine the interaction between *C. hirtus* and *S. sphagnetorum*, both in their intact form and after artificial removal of rhabdoid gland content; (2) assess the ability of the mucus secretion to neutralize the toxic activity of *C. hirtus* toxicysts; and (3) investigate the basic composition of the mucous material produced by *S. sphagnetorum.*

## 2. Materials and Methods

### 2.1. Organisms and Culture Methods

*Stenostomum sphagnetorum* (Platyhelminthes: Turbellaria) (approx. 1400 × 110 μm^2^) [5] was cultured in Synthetic Medium for *Blepharisma* (SMB) [13] and fed with the ciliate *Paramecium multimicronucleatum* (clone TL-2).

*Coleps hirtus* (clone PC-4) (approx. 50 × 30 μm^2^) [9] was cultured in SMB and fed with the flagellate *Chlorogonium elongatum* Dangeard, 1899, cultivated as described in [14] or in Jaworski’s Medium (JM) solution.

### 2.2. Preparation of Secretion-Deprived S. sphagnetorum

The technique employed to induce maximal mucus secretion in *S. sphagnetorum* by lysozyme treatment is not specific to the rhabdoid glands; rather, it elicits an acute stress response in the organism, to obtain the instantaneous secretion of the protective mucus capsule. This treatment, as described in [3], triggers the discharge of mucus secretions without harming the organisms. To obtain secretion-deprived (SD) *S. sphagnetorum*, approximately 100 individuals/mL were mixed 1:1 with a 250 μg/mL lysozyme solution (Sigma-Aldrich, St. Louis, MO, USA, from chicken egg white) in SMB. Mucus was often visible as a capsule surrounding the organism under a stereomicroscope. After 5 min of incubation, the mixture was centrifuged at 45× *g* for 3 min just enough to pellet the catenulids but not the mucus. The pellet was washed thoroughly and resuspended in fresh SMB for 2 h before use in experiments as secretion-deprived (SD) specimens.

### 2.3. Isolation of Toxicyst Discharge of C. hirtus

Toxicyst discharge (TD) from *C. hirtus* was isolated following the cold-shock induction protocol described in [9]. Briefly, a dense culture of ciliates (approximately 60,000 cells/mL) was rapidly mixed with ice-cooled SMB buffer in a 1:5 (*v*/*v*) ratio. The mixture was incubated at 0 °C for 3 min to induce extrusome discharge. Following induction, the suspension was gently centrifuged at 55× *g* for 4 min to pellet the cells. The resulting supernatant, containing the released toxicysts, was carefully collected and subjected to vacuum centrifugation to remove the solvent. The dried extract was then weighed and stored at −20 °C until further use.

### 2.4. Liquid Chromatography–Mass Spectrometry

Samples of secretion-containing capsules were resuspended in 200 μL of ethanol, sonicated at 4 °C for 15 min (Sonorex Super, Bandelin electronics, Berlin, Germany), and centrifuged at 4000× *g* for 10 min at room temperature. The procedure was repeated 3 times, and the supernatant was removed in a rotatory evaporator and resuspended in 500 μL of methanol. Aliquots of 10 μL were injected and analyzed in negative ionization mode on a Hewlett–Packard Model 1100 Series liquid chromatograph (Hewlett–Packard Development Company, L.P., Palo Alto, CA, USA) coupled both to a Bruker Esquire-LC quadrupole Ion Trap Mass Spectrometer (IT-MS) equipped with an Electrospray Ionization (ESI) source (Bruker Optik GmbH, Ettlingen, Germany) and to a photo diode-array detector (DAD) (Agilent Technologies, Milan, Italy). Chromatographic separation was carried out on a Zorbax Eclipse XDB-C18 column (150 × 4.6 mm, 3.5 μm; Hewlett Packard, Palo Alto, CA, USA), with a linear gradient of solvent A (methanol: water 7:3, containing 12 mM ammonium acetate) and solvent B (methanol containing 12 mM ammonium acetate) from 0% B to 100% B in 30 min, at a constant flow rate of 1.0 mL/min. Final conditions were kept for at least 30 min. Mass range was 50–1200 *m*/*z*, and high-voltage capillary was set at −4000 V. DAD was operated at 205, 215, 254, 574 and 590 nm. High-resolution MS was conducted using same chromatographic setup on an Orbitrap Fusion™ Tribrid™ Mass Spectrometer (Thermo Fisher Scientific, Waltham, MA, USA).

### 2.5. Analysis of the Content of Glandular Secretion of S. sphagnetorum

After the lysozyme treatment of a massive culture of *S. sphagnetorum*, the empty capsules composed of mucus material were removed by a micropipette, extensively washed in SMB and processed for SDS-PAGE analysis, or used both for periodic acid-Schiff (PAS) staining and for Alcian blue staining. The electrophoresis analysis of the glandular secretion was performed on 12.5% SDS gels essentially according to Laemmli [15] and stained with Coomassie blue R-250.

The PAS staining or Alcian blue staining was used for the detection of neutral and acid glycosaminoglycans in the glandular secretion, respectively. For the PAS staining, the empty capsules, or the specimens together with their secretion, were fixed on microscope slides for 1 min, at room temperature, with the fixative solution (10% *v*/*v* formaline, 90% *v*/*v* ethanol). Slides were rinsed for 1 min with slowly running tap water and exposed in periodic acid solution (Sigma-Aldrich, St. Louis, MO, USA) for 5 min, washed several times and exposed to Schiff’s reagent (Sigma-Aldrich, St. Louis, MO, USA) for 7 min. After this procedure, the slides were washed in running tap water for 5 min and counterstained in hematoxylin solution (Sigma-Aldrich, St. Louis, MO, USA) for 90 s. Finally, the slides were washed for 30 s, dehydrated and mounted with Canada balsam.

For the detection of acid glycosaminoglycans, an Alcian blue solution was prepared at pH 2.5 by dissolving 1 g of Alcian Blue (8GX, Sigma-Aldrich, St. Louis, MO, USA) in 100 mL of 3% acetic acid solution.

As for PAS staining, the capsules were fixed for 1 min on microscope slides with the formalin–ethanol fixative solution (Sigma-Aldrich, St. Louis, MO, USA). Slides were then placed in 3% acetic acid for 5 min, stained with Alcian blue solution at pH 2.5 for 30 min, rinsed in running tap water for 1 min, dehydrated and mounted with Canada balsam.

For the detection of protein components, the glandular secretion of *S. sphagnetorum* was stained with Coomassie blue R-250. Briefly, five to ten specimens of the microturbellarian were placed on microscope slides and 10 µL of saturated aqueous picric acid solution was added to induce glandular secretion. This procedure was established in order to avoid contamination with lysozyme, which could have interfered with Coomassie staining. Excess fluid was removed with a micropipette and cold formalin/ethanol (10/90%, *v*/*v*) fixative solution was then added for 1 min at room temperature. Microscope slides were washed for 1 min with distilled water, stained with Coomassie solution (0.1% *w*/*v* Coomassie blue R350, 20% *v*/*v* methanol, and 10% *v*/*v* acetic acid) for 1 h, and incubated in destaining solution (10% *v*/*v* acetic acid, 20% *v*/*v* methanol, in water) for 30 min. Slides were finally rinsed in distilled water for 1 min, dehydrated and mounted with Canada balsam.

### 2.6. Toxicity Test

For dose–response experiments, triplicate samples of ten control or treated *S. sphagnetorum* specimens (SD) were placed in depression slides containing 250 μL of SMB with increasing concentrations of either the tested fatty acid or toxicyst discharge from *C. hirtus*. The slides were maintained in a dark, moist chamber, and organism viability, defined by normal morphology and locomotion, was assessed after 1 h and 24 h. Median lethal concentrations (LC_50_) were determined by fitting concentration–survival data to a nonlinear regression model using GraphPad Prism 6 (GraphPad Software, San Diego, CA, USA), following the procedure described in [3] and applying a 95% confidence interval.

### 2.7. Chemicals

Dodecanoic acid (C12:0), myristic acid (C14:0, 99%), oleic acid (C18:1, pure), palmitic acid (C16:0), palmitoleic acid (C16:1, 99%), and stearic acid (C18:0, 98%) were purchased from Carlo Erba Reagents (Cornaredo, Italy). Each fatty acid was dissolved in ethanol (5 mg/mL) at 60 °C under continuous stirring. The resulting solutions were stored at room temperature and subsequently diluted first in 70% ethanol and then in SMB immediately prior to use in experiments. Notably, a stable solution of stearic acid in SMB could not be achieved.

## 3. Results

### 3.1. Predator–Prey Interactions Between S. sphagnetorum and C. hirtus

Approximately 200 cells of *C. hirtus* were mixed with 5 specimens of *S. sphagnetorum* in 500 μL of SMB and the interactions were initially investigated under a stereomicroscope.

**Figure 2:**
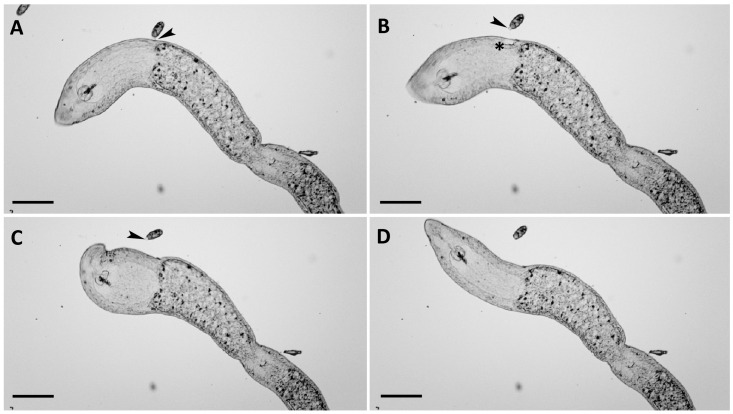
The predator–prey interaction between *C. hirtus* and *S. sphagnetorum*. (**A**) A cell of *C. hirtus* contacts a specimen of *S. sphagnetorum* with its anterior part, and immediately the discharged mucous material (arrowhead) emerges to separate the two organisms. (**B**) The *C. hirtus* stops swimming and starts eating the still noticeable mass of mucous material (arrowhead). The asterisk indicates the body part of *S. sphagnetorum* involved in the discharge of the mucus, visible as a slight depression in the body shape. (**C**) A few seconds later (1–3), the *C. hirtus* continues to eat the discharged mucus. (**D**) Soon after, the predator has finished eating the discharged material while the *S. sphagnetorum* swims away unharmed. Micrographs extracted from a film clip. Scale bars = 100 µm.

Two distinct types of interactions were observed: type I occurred when the anterior region of a *C. hirtus* cell made contact with a specimen of *S. sphagnetorum*. While initial encounters involved brief and transient contacts between the two species, sustained interactions became clearly visible after 3–5 min, when a more prolonged contact between predator and prey was evident. During this phase, the microturbellarian exhibited increased swimming speed, while *C. hirtus* temporarily ceased movement, followed by its eventual detachment from the *S. sphagnetorum* body (Figure 2A,D). This behavior was often accompanied by feeding activity in the ciliate (Figure 2B,C; Video S1), suggesting that *C. hirtus* may have consumed secretions released by *S. sphagnetorum*. In this kind of interaction, *S. sphagnetorum* seems to be capable of defending itself against the attacks of *C. hirtus*.

**Figure 3:**
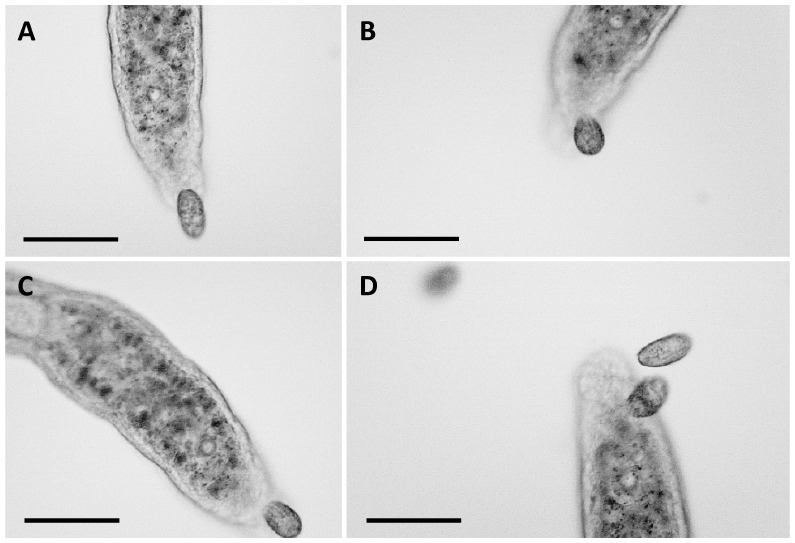
The predator–prey interaction between *C. hirtus* and secretion-deprived (SD) specimens of *S. sphagnetorum*. (**A**) A cell of *C. hirtus* contacts an SD specimen of *S. sphagnetorum* with its anterior part. (**B**) A few seconds later (3–9), the *S. sphagnetorum* increases its swimming speed while the predator remains attached to its body. (**C**) subsequently (10–15 s) the cell of *C. hirtus* remains attached to the body of the prey for a prolonged time. (**D**) more *C. hirtus* cells approach the prey. Micrographs extracted from a film clip. Scale bars = 100 µm.

Type II interaction occurred when the anterior part of *S. sphagnetorum* contacted a cell of *C. hirtus* which was entirely ingested by the flatworm given the very small size of the ciliate. In this type of interaction *S. sphagnetorum* acts as a predator of *C. hirtus*, however, this behavior has not been deeply investigated as it does not involve the utilization of glandular secretions of *S. sphagnetorum*. To assess the role of *S. sphagnetorum*’s glandular secretions in type I defensive behavior, additional observations were conducted using 5 SD specimens of *S. sphagnetorum* mixed with ~200 cells of *C. hirtus* in 500 μL of SMB. In this mixture, effective and prolonged contacts were observed within a few minutes, often accompanied by an increase in prey swimming speed (Figure 3). However, unlike the interactions involving untreated *S. sphagnetorum*, *C. hirtus* cells often remained attached to their prey for extended periods, with a gradual reduction in the prey’s swimming speed (Figure 3). In some cases, additional *C. hirtus* cells joined the attack on the same prey (typically targeting the tail region), ultimately immobilizing it at the bottom of the depression slide. The prey was visibly injured by the predators, and in some instances, shortening of the caudal section was observed. Occasionally, the prey managed to survive and regenerated its damaged parts (in about 24 h), but in other instances, it was completely consumed within a few hours. It is interesting to note that type II interaction never occurred using SD specimens of *S. sphagnetorum* within 30 min of observation.

To quantitate these findings, nine parallel experiments were conducted, each involving around 500 cells of *C. hirtus* and one specimen of *S. sphagnetorum*, prepared in multiple depression slides with 250 μL of SMB. The experiments were performed using both untreated and SD specimens of *S. sphagnetorum*. After 6 h, all untreated specimens of *S. sphagnetorum* survived (1, *n* = 9), whereas some of the SD specimens disappeared. Specifically, the number of surviving SD specimens was reduced to 5 out of 9 (0.56 ± 0.18, *n* = 9). These results strongly suggest that the glandular secretions of *S. sphagnetorum* play a relevant role in defending the microturbellarian against predation by *C. hirtus*.

### 3.2. Toxicity Evaluation of TD of C. hirtus and Fatty Acids on S. sphagnetorum

**Figure 4:**
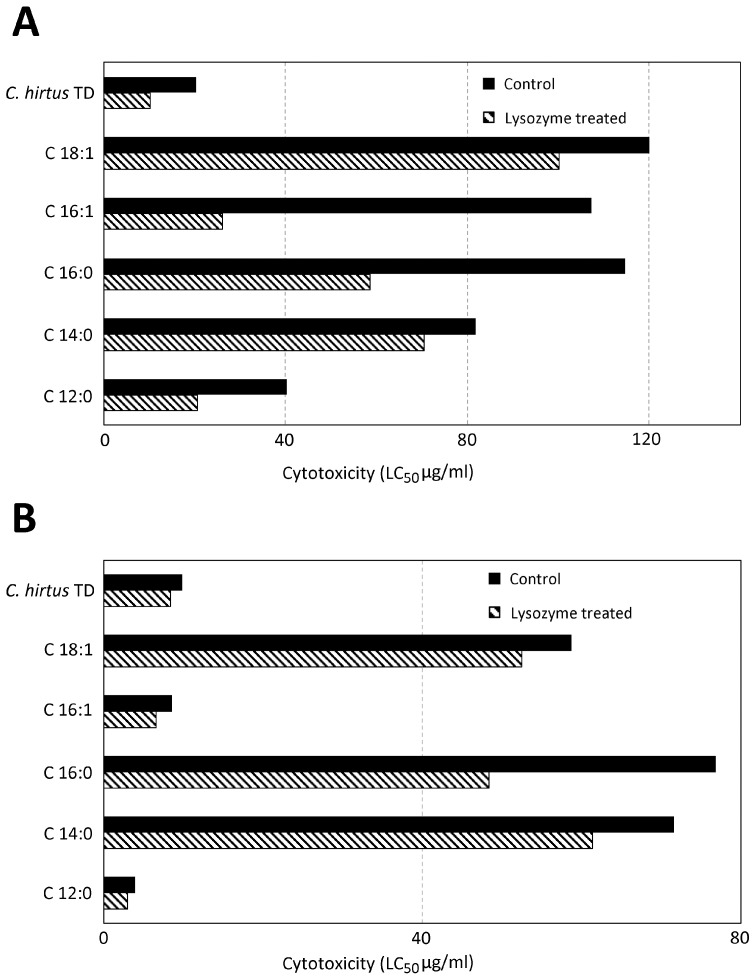
Comparison of the cytotoxic effects of the discharge of *C. hirtus* (TD) and the five more representative free fatty acids included in the discharge, on control or SD specimens of *S. sphagnetorum.* Viability was assessed after 1 (**A**) or 24 h (**B**) of incubation and the LC_50_ values were obtained by nonlinear regression analysis of three independent experiments with the 95% confidence limits calculated using GraphPad Prism 6 software.

To investigate the protective role of mucus secretion in *S. sphagnetorum* against the toxins used by the predatory ciliate *C. hirtus* for predation, we performed a set of dose–response experiments using either control or SD specimens of *S. sphagnetorum*. These assays tested the toxicity of the complete toxic discharge (TD) of *C. hirtus* as well as 5 representative free fatty acids (FFA) that compose the TD [9]. Toxicity was assessed after 1 and 24 h of exposure, with results summarized in Figure 4. It appears that each FFA analyzed exhibits toxic activity against both control and SD specimens of *S. sphagnetorum*, however, SD organisms consistently show significantly higher sensitivity to each compound. The most toxic substances identified were dodecanoic acid and the total discharge of *C. hirtus*. Overall, the data confirm the protective role of *S. sphagnetorum* mucus secretion against the toxins used by the predatory ciliate.

### 3.3. Characterization of the Main Components of the Secretion of S. sphagnetorum

**Figure 5:**
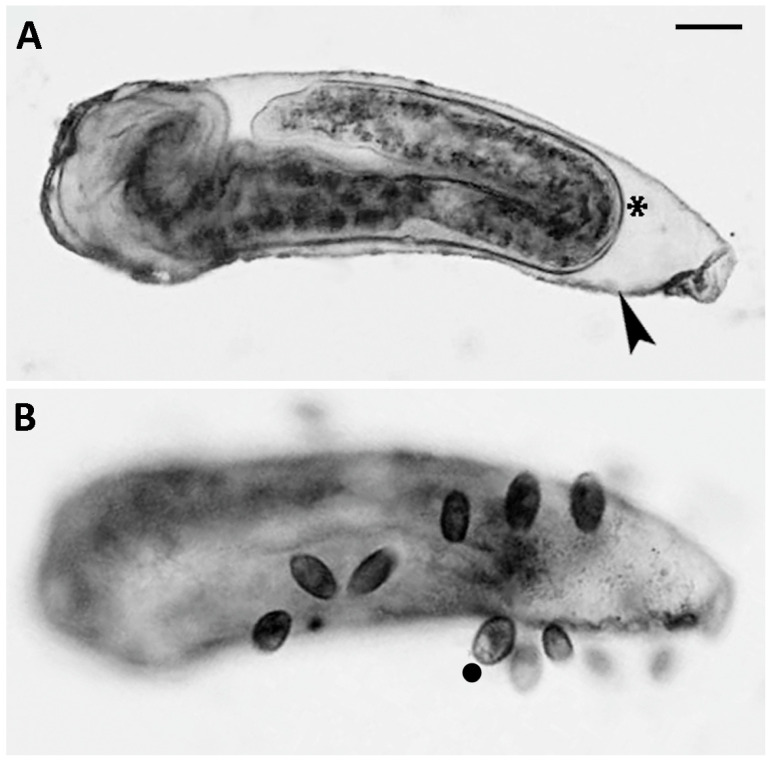
(**A**) Formation of a capsule after the lysozyme treatment in *S. sphagnetorum* (arrow head). Often, the specimen remained alive inside the capsule (asterisk) for several minutes and did not escape. (**B**) The same individual of *S. sphagnetorum* after the addition of about 50 cells of *C. hirtus* (filled circle) which attack and consume the capsule secreted by *S. sphagnetorum*. Scale bar = 100 µm.

The first approach used for the characterization of the mucus components of *S. sphagnetorum* was the liquid chromatography–electrospray ionization-mass spectrometry technique (LC-ESI-MS). However, samples of secretion-containing capsules proved to be substantially insoluble in the chromatographic buffer, even after extensive sonication, thus making it impossible to separate their components and perform mass spectrometry analysis. Therefore, in order to obtain at least preliminary data on the main components of the mucus, we subjected the capsules secreted (Figure 5A) by the worm to basic analyses: PAS, Alcian blue and Coomassie blue stainings, SDS-PAGE.

**Figure 6:**
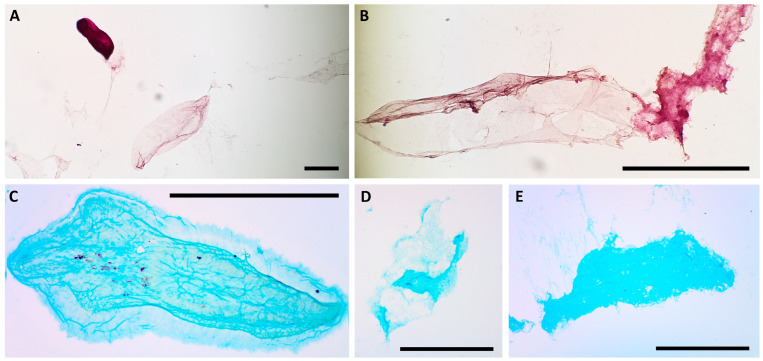
Periodic acid-Schiff (PAS) staining system (**A**,**B**) used to reveal the presence of neutral glycosaminoglycans in the capsules (arrowhead) secreted by *S. sphagnetorum.* (**A**) Asterisk indicates a fixed and contracted specimen of *S. sphagnetorum.* The alcian blue staining (**C**–**E**), is used to reveal the presence of acid glycosaminoglycans. In PAS staining, neutral glycosaminoglycans appear magenta, whereas in alcian blue staining acid glycosaminoglycans appear faint blue. (**C**) A whole capsule; (**D**,**E**) Capsule fragments. Scale bars = 200 µm.

PAS staining produced a red-to-pink coloration of capsules, indicating the presence of neutral glycosaminoglycans (Figure 6A,B). Furthermore, the presence of acid glycosaminoglycans was suggested by Alcian blue staining (Figure 6C–E).

**Figure 7:**
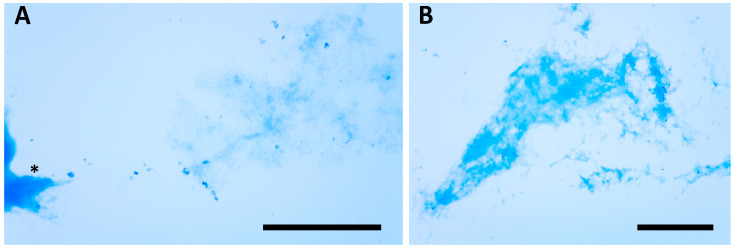
Coomassie blue staining used to reveal the presence of proteins into the capsule’s secretion of *S. sphagnetorum.* (**A**) Asterisk indicates a fixed and contracted specimen of *S. sphagnetorum*. (**B**) an empty and fragmented capsule. Scale bars = 200 µm.

SDS-PAGE of secretion-containing capsules revealed no detectable protein components, both under reducing and non-reducing conditions, at least within the molecular weight range of 250–10 kD. On the other hand, the fact that the capsules apparently did not dissolve in SDS, with or without betamercaptoethanol, during sample preparation suggested the need for an alternative approach to investigate the presence of proteins. Therefore, we used Coomassie blue staining for protein detection, taking care to induce glandular secretion in the absence of lysozyme, which could have interfered with the staining. In this case, in contrast to the electrophoretic analysis, the Coomassie blue staining of the glandular secretions indicated the presence of proteins in the capsules, in addition to the mucopolysaccharide components (Figure 7).

These results are consistent with the behavior of *C. hirtus*, which, during its interaction with *S. sphagnetorum*, interrupts its attack to consume the glandular secretion. Moreover, we observed that *C. hirtus* could attack the capsule when introduced into a culture of *S. sphagnetorum* that had secreted capsules following lysozyme treatment (Figure 5B).

## 4. Discussion

Flatworms, comprising approximately 22,500 species worldwide [16], remain chemically understudied despite employing fascinating offense/defense mechanisms mainly mediated by mucus secretions of epidermal and subepidermal gland cells. Among these, two main types are represented by rhabdite glands, containing rod-shaped secretions with a lamellated core, and rhabdoid glands, containing electron-opaque, spherical to ovoid secretions [17]. In particular, functions of rhabdite glands secretion (mucus) that have been widely described include protection against viruses, bacteria, fungi, predators, and wound sealing [3,16,18,19]. In many species, the frontal glands can secrete large amounts of mucus, facilitating planarian locomotion or prey immobilization [16,20]. Some species of flatworms are also capable of adding toxic molecules, such as tetrodotoxin, to the secretions of the epidermal glands primarily for chemical defense against predators [21]. In any case, even if many of the functions of rhabdite gland secretion have been studied, its chemical composition remains unknown, except for a partial characterization of its protein content [22].

In contrast to the well-studied rhabdite glands, very little is known about the function of the mucus produced by the rhabdoid glands, such as those found in the genus *Stenostomum* [3,5,23], and virtually nothing is known about its chemical composition [24]. In this paper, we partially fill this knowledge gap by describing the defensive and protective function of the mucus secretion of *S. sphagnetorum* against the predator ciliate *C. hirtus* and its toxins, and, for the first time, partially characterizing its molecular composition [25].

### 4.1. Predator–Prey Interactions

The results of this study demonstrate that *S. sphagnetorum* can defend itself against the predatory ciliate *C. hirtus*, primarily through the discharge of a mucous substance from its secretory glands, which serves as the key component of its antipredator behavior. As in the case against the raptorial ciliate *D. margaritifer*, this defensive function is essentially mechanical, since the secretion immediately separates the predator from the prey and prompts the predator to interrupt its attack to consume the discharged secretion. This behavior is further confirmed by the observations that *C. hirtus* cells are also capable of attacking and feeding on the capsules secreted by *S. sphagnetorum* following lysozyme treatment. The effectiveness of this defense mechanism is supported by our findings, as *S. sphagnetorum* specimens artificially deprived of mucus secretions are significantly more vulnerable to predation by *C. hirtus* compared to untreated catenulids. Additionally, SD specimens were observed to be temporarily unable to counterattack and feed on *C. hirtus*, unlike their untreated counterparts. While this behavior warrants further investigation, it is plausible to speculate that *S. sphagnetorum*, known to selectively prey on organisms based on their toxicity [5], may employ an additional defensive strategy. Specimens that are temporarily unable to discharge their glandular secretions might actively avoid interactions with toxin-secreting organisms, thereby mitigating the potential risks associated with their impaired defensive state.

### 4.2. Toxicity Tests

The results obtained after the exposure of both untreated and SD specimens of *S. sphagnetorum* to five of the more representative toxic FFA of *C. hirtus* apparently resemble those previously published that describe the effect of the toxin of *D. margaritifer* on the microturbellarian [3]. Here, as in the case described in the previous report, SD specimens proved to be significantly more sensitive to protozoan toxins, although the chemical nature of the toxins was substantially different. In fact, while the toxic action of *Dileptus* is mediated by a protein enzyme (acid phosphatase) [26], in the case of *Coleps*, toxicity is determined by a mixture of fatty acids, suggesting a broad spectrum of chemical protection provided by secretion of the rhabdoid glands of *Stenostomum*. In addition, the increased resistance of untreated individuals of *S. sphagnetorum* to various toxins also suggests that, under normal conditions, there must be a constitutive basal secretion of mucus constantly lining the worm’s surface, perhaps facilitating locomotion and also providing a first line of defense against possible pathogens, predators, and toxic molecules, as extensively documented for Plathyhelminthes, Rhabditophora [17,18,22].

### 4.3. Chemical Characterization

To the best of our knowledge, no studies have yet reported data on the chemical and structural characterization of the mucus produced by the rhabdoid glands present in Catenulida, whereas several studies have investigated the mucus secretions of the rhabdite glands. The latter have in some cases revealed the presence in the mucous secretions of sulphated glycosaminoglycans [27], as well as dozens of proteins, including some with antioxidant activity, others with antibiotic activity [22,25]. We also performed a preliminary characterization of the mucous secretion from the rhabdoid glands of *Stenostomum*, using specific dyes to detect glycosaminoglycans and proteins.

As indicated in the Results Section, both neutral and acidic glycosaminoglycans were detected in the secretion-containing capsules, while the presence of proteins was only observed through Coomassie staining. In fact, the fractionation of protein components by electrophoresis analysis was not possible, likely due to the presence of high molecular weight aggregates that require further investigation to better understand its biological functions.

Overall, based on the results obtained in this work, it can be stated that the mucus secretion produced by *Stenostomum* can exist in at least two different physical states: a “basal” state and an “aggregated” state. It can be hypothesized that, in the basal state, the mucus constantly coats the entire surface of the worm, facilitating its gliding on the substrate and protecting it from potential toxins, such as those produced by *Coleps*; the aggregated state, which leads to the formation of protective capsules, is instead activated in response to strong environmental stimuli, such as a predator attack. In both cases, it is possible that both glycosaminoglycans and proteins are synergistically involved, although the data currently available do not allow us to distinguish the specific functions (mechanical and chemical) of the individual components.

## 5. Conclusions

Taken as a whole, the data reported in this study indicate that *S. sphagnetorum* adopts a defensive mechanism against ciliate predators by means of the mucus secretion of the rhabdoid glands. These findings would not only broaden our understanding of the metabolites produced by Catenulidae, thereby shedding light on predator–prey interactions among microinvertebrates, but could also have potential applications in medicine and pharmacology, as is increasingly the case with many classes of natural compounds [28,29,30,31,32].

As an example, 119 proteins homologous to those found in human tears and mucus secretions have been identified in the mucus of *Schmidtea mediterranea*, suggesting the planarian as a suitable animal model for the study of human tears properties [22]. Of course, in the case of *Stenostomum*, further studies will be necessary to obtain a functional and structural characterization of all the protein components of the mucus, thereby assessing any potential applications.

## Data Availability

The data presented in this study are available upon reasonable request from the corresponding authors. The data are not publicly available due to privacy.

## Supplementary Materials

The following supporting information can be downloaded at: https://www.mdpi.com/article/10.3390/biology14091253/s1, Video S1: predator–prey interaction.

## Abbreviations

The following abbreviations are used in this manuscript:

SMB	Synthetic medium for *Blepharisma*
SD	Secretion-deprived
TD	Toxicyst discharge
PAS	Periodic acid–Schiff
